# Long-term Safety and Effectiveness of Cold-Crosslinked Hyaluronic Acid Fillers: Multicenter, Randomized, Controlled, Double-Blind Study

**DOI:** 10.1093/asj/sjaf080

**Published:** 2025-05-16

**Authors:** Michael S Kaminer, Rui L Avelar, Leslie Baumann, Valerie Callender, Steven H Dayan, Jeremy B Green, Steven K Grekin, Sebastien Guyon

## Abstract

**Background:**

Evolysse Form (EVL_F_) and Evolysse Smooth (EVL_S_) (Symatese, Chaponost, France) are new hyaluronic acid fillers created using an innovative cold-crosslinking process.

**Objectives:**

The authors of this study aim to collect safety and effectiveness data on new cold-crosslinked fillers to support the US approval for the correction of moderate-to-severe dynamic facial wrinkles and folds.

**Methods:**

In this randomized, controlled, split-face study, 140 patients with moderate-to-severe nasolabial folds (NLFs) received a cold-crosslinked filler in 1 NLF (EVL_F_ = 70, EVL_S_ = 70) and a traditionally crosslinked filler, Restylane-L (RES_L_), in the contralateral fold and were followed through 12 months with an optional retreatment at that time point and subsequent 3 months of safety follow-up.

**Results:**

The primary endpoint of mean Wrinkle Severity Rating Scale change from baseline to Month 6 as rated by the photographic review panel demonstrated noninferiority and statistical superiority for the cold-crosslinked fillers. Blinded evaluator Wrinkle Severity Rating Scale assessments showed a mean change from baseline that was statistically significantly better than RES_L_ for EVL_F_ at all visits through 12 months and for EVL_S_ at 6 and 9 months. Most patients were responders on the Global Aesthetic Improvement Scale throughout the study, according to ratings by blinded evaluators, treating investigators, and patients. The FACE-Q appraisal of NLFs’ overall mean score showed significant improvement from baseline (*P* < .0001) at all time points through Month 12 for all treatment groups. All treatments were well tolerated.

**Conclusions:**

The new cold-crosslinked fillers were shown to be safe and effective for correction of NLFs, with results lasting for 1 year.

**Level of Evidence: 1 (Therapeutic):**

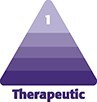

Hyaluronic acid (HA) fillers are widely used in aesthetic treatments because of their biocompatibility. Crosslinking of the HA molecules is a necessary step to create fillers with appropriate mechanical properties and duration in the skin, and the BDDE (1,4-Butanediol diglycidyl ether) crosslinker enables the HA molecules to resist enzymatic and oxidative degradation. The crosslinking step in manufacturing fillers is traditionally done at an elevated pH and in a heated state, and the highly alkaline conditions activate the BDDE epoxide rings, which leads to the crosslinking of the HA by forming covalent bonds. However, in this caustic environment, HA fragmentation can occur, further exacerbated by thermal energy (eg, heat) of the reaction conditions.^1,2^ Evolysse Form (EVL_F_) and Evolysse Smooth (EVL_S_) (Symatese, Chaponost, France) are new HA fillers created using an innovative cold-crosslinking process (Cold-X Technology). Performing the crosslinking process in a cold state helps to better preserve the long HA chains and requires less BDDE to achieve the targeted rheological properties. Less fragmentation of the HA chains aims to improve safety as well as the performance and duration of the fillers.

The purpose of this pivotal study was to collect safety and effectiveness data on the new cold-crosslinked injectable HA gels, EVL_F_ and EVL_S_, which supported approval by the US FDA for the correction of moderate-to-severe dynamic facial wrinkles and folds.

## METHODS

### Study Design

This randomized, controlled, split-face study was conducted at 6 sites in the United States, with patients and evaluators being blinded to which HA filler was injected in each nasolabial fold (NLF), and investigators who performed the treatments being unblinded. Patients were randomized using an electronic data capture system in a 1:1:2 ratio to receive one of the EVL fillers in 1 NLF and Restylane-L (RES_L_; Q-Med AB, Sweden) in the other (side of the face for each treatment was randomized), and randomization was stratified by Fitzpatrick skin type (I-III and IV-VI) and investigational site to ensure balance between treatment arms. Follow-up visits occurred 2 and 6 weeks after initial treatment as well as at 3, 6, 9, and 12 months. Patients electing to receive retreatment at 12 months were followed at 2 weeks and 3 months after that for safety only.

The study was reviewed and approved by WCG IRB and was registered at www.clinicaltrials.gov (NCT0532541; https://clinicaltrials.gov/study/NCT05325411?term=NCT05325411&rank=1). Written consent was provided, by which the patients agreed to the use and analysis of their data. All study visits occurred between May 2022 and March 2024.

### Patients

Patients were required to be aged 22 and older and have moderate or severe NLFs on the Wrinkle Severity Rating Scale (WSRS) as scored live by a blinded evaluator. Washout periods were 2 weeks for topical treatments (prescription wrinkle treatments, steroids, and self-tanners), 3 months for noninvasive skin-tightening treatments, 6 months for ablative or nonablative dermal resurfacing procedures, and 12 months for neurotoxin injections below the orbital rim and nonpermanent facial fillers. Patients with any history of permanent facial fillers were excluded.

### Treatment

Patients received a cold-crosslinked filler (EVL_F_ or EVL_S_) in 1 NLF and a traditionally crosslinked filler (RES_L_) approved for NLF treatment in the contralateral NLF. HA concentration was 22 mg/mL for EVL_F_ and 20 mg/mL for EVL_S_ and RES_L_, and all 3 fillers were crosslinked with BDDE and contained 0.3% lidocaine to minimize injection pain. Initial treatment and optional touch-up 2 weeks later were with the randomized fillers in each respective NLF, and optional retreatment at 12 months plus accompanying optional touch-up were with the EVL fillers in both NLFs as needed. Treatments were performed according to each product's instructions for use, which included linear threading (antegrade and retrograde), crosshatching, and serial puncture as options for injection techniques. Injection volume was determined by the treating investigators.

### Assessments

The validated 5-point photonumeric WSRS was used by blinded evaluators at screening and all follow-up visits from 6 weeks through 12 months to rate NLFs as absent, mild, moderate, severe, or extreme.^3^ An independent photographic review panel of 3 physicians performed assessments with the same scale using photographs from screening and Month 6 visits.

Blinded evaluators, treating investigators, and patients all performed assessments on the 5-point Global Aesthetic Improvement Scale (GAIS: very much improved, much improved, improved, no change, and worse) by comparing screening photographs to live appearance at all follow-up visits from 6 weeks through 12 months. Patients completed the validated FACE-Q appraisal of NLF questionnaire at screening and all follow-up visits from 6 weeks through 12 months to rate how bothered they were by various aspects of their NLFs on a 4-point scale (not at all, a little, moderately, and extremely).^4^ At the same time points, patients used screening photographs for comparison and completed the Aesthetic Appearance Questionnaire to rate treatment outcomes on 4-point satisfaction scales (very satisfied, somewhat satisfied, somewhat dissatisfied, and very dissatisfied) or 5-point agreement scales (strongly agree, agree, neither agree nor disagree, disagree, and strongly disagree). Immediately after each study treatment and at 10, 20, and 30 min, patients rated pain in each NLF in half-point increments on a scale ranging from 0 to 10. For all effectiveness assessments, each NLF was rated individually.

For 30 days after each study treatment, patients completed daily diaries to rate the intensity of common treatment responses as mild, moderate, or severe. At all study visits, adverse events (AEs) were collected, and Snellen eye examinations and ocular movement assessments were performed (at least 30 min after study injections at treatment visits).

### Statistics

The primary endpoint was the mean WSRS change from baseline to Month 6 as rated by the photographic review panel for the modified intent-to-treat population and analyzed with a Wilcoxon signed-rank test. Secondary endpoints were evaluated for the modified intent-to-treat population and included WSRS responder rates (NLFs with at least 1-grade improvement), according to photographic review panel and blinded evaluators (95% CIs); WSRS mean change (Wilcoxon signed-rank test) and superiority or equivalence as assessed by blinded evaluators; GAIS improvement as assessed by blinded evaluators, treating investigators, and patients (95% CIs); FACE-Q appraisal of NLF improvement vs baseline (paired *t* test); and pain scale (95% CIs). An additional endpoint was the Aesthetic Appearance Questionnaire (95% CIs). Analyses of WSRS mean change and responder rate at Month 6 were performed with 95% CIs for the subgroups of sex, race, ethnicity, age group, Fitzpatrick skin type, injection volume, baseline WSRS score, and touch-up status.

Common treatment responses and AEs were coded to MedDRA terms and summarized with descriptive statistics. Subgroup analyses of common treatment responses and AEs were performed by sex, race, ethnicity, age group, Fitzpatrick skin type, injection volume, baseline WSRS score, touch-up status, and needle size.

Sample size was calculated using statistical software PASS version 16.0.4 and determined that a minimum of 25 patients were needed in each study arm to achieve at least 95% power with a noninferiority margin of 0.5 points for the primary endpoint. However, the study sought to enroll 70 patients in each arm to collect adequate safety data and account for potential attrition.

## RESULTS

### Patient and Treatment Characteristics

A total of 141 patients were randomized, with 1 patient in the EVL_F_ arm discontinuing before treatment, resulting in 70 treated patients in each study arm (Table 1). The majority of treated patients finished follow-up through the entirety of the study, with 87.1% (61/70) in the EVL_F_ arm and 92.9% (65/70) in the EVL_S_ arm completing the study. Most patients were female and white, and all Fitzpatrick skin types were represented. All patients had each NLF rated as moderate or severe on the WSRS as required in the study eligibility criteria.

**Table 1.** Patient Disposition and Demographics/Baseline Characteristics

	EVL_F_ arm (*n* = 70) *n* (%)	EVL_S_ arm (*n* = 70) *n* (%)
Randomized (population)	71	70
Treated (modified intent-to-treat and safety populations)	70	70
Per-protocol population	62 (88.6)	62 (88.6)
Discontinued after treatment	9 (12.9)	5 (7.1)
Primary reason for discontinuation		
Withdrawal by patient	6 (8.6)	3 (4.3)
Lost to follow-up	3 (4.3)	1 (1.4)
Adverse event (knee replacement)	0	1 (1.4)
Female	66 (94.3)	65 (92.9)
Age, median (range)	59.5 (25-83)	57 (31-84)
Race		
White	51 (72.9)	50 (71.4)
Black or African American	14 (20.0)	19 (27.1)
Asian	2 (2.9)	0
Other/multiple	3 (4.3)	1 (1.4)
Ethnicity: Hispanic or Latino	21 (30.0)	18 (25.7)
Fitzpatrick skin type		
I	2 (2.9)	1 (1.4)
II	19 (27.1)	16 (22.9)
III	22 (31.4)	22 (31.4)
IV	16 (22.9)	14 (20.0)
V	9 (12.9)	11 (15.7)
VI	2 (2.9)	6 (8.6)
Baseline WSRS score		
Both NLFs moderate	33 (47.1)	39 (55.7)
Both NLFs severe	26 (37.1)	19 (27.1)
EVL severe, RES moderate	6 (8.6)	7 (10.0)
EVL moderate, RES severe	5 (7.1)	5 (7.1)

EVL_F_, Evolysse Form; EVL_S_, Evolysse Smooth; NLF, nasolabial fold; RES, Restylane; WSRS, Wrinkle Severity Rating Scale.

Of the 70 patients in the EVL_F_ arm, 51 received touch-up with EVL_F_ and 52 with RES_L_, with a mean volume injected for initial and touch-up treatments combined of 1.2 mL for EVL_F_ (range, 0.2-3.0 mL) and 1.3 mL for RES_L_ (range, 0.5-3.2 mL). Retreatment was performed for 44 of the EVL_F_ NLFs and 47 of the control NLFs, with a mean total EVL_F_ volume injected for retreatment and retreatment touch-up treatments combined of 0.8 mL for each NLF (range, 0.02-1.9 mL).

Of the 70 patients in the EVL_S_ arm, 54 received touch-up in each NLF, with a mean volume injected for initial and touch-up treatments combined of 1.0 mL for EVL_S_ (range, 0.4-1.9 mL) and 1.2 mL for RES_L_ (range, 0.5-3.0 mL). Retreatment was performed for 44 NLFs on each side, with a mean total EVL_S_ volume injected for retreatment and retreatment touch-up treatments combined of 0.6 mL for each NLF (range, 0.05-1.4 mL).

The needle size was 27 G (54.3%) or 30 G (45.7%) for EVL_F_ injections, 30 G for EVL_S_ injections, and 29 G for all control injections. The most common injection techniques for all NLFs were retrograde linear threading (82.9%-85.7%) and crosshatching (38.6%-40.0%).

### Effectiveness

**Figure 1.**
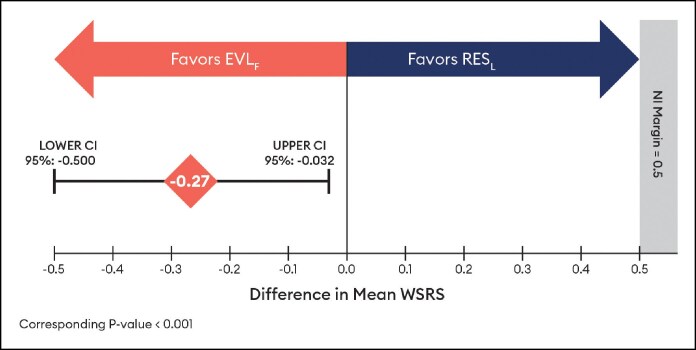
Wrinkle Severity Rating Scale difference in mean change at Month 6 by independent photographic review panel for Evolysse Form (EVL_F_) arm.

For the EVL_F_ primary endpoint (*n* = 62), the absolute difference in the mean WSRS change from baseline to Month 6 for EVL_F_ vs RES_L_ was −0.27 (95% CI, −0.500 to −0.032), favoring EVL_F_, and the upper bound of the 95% CI was <0.5 threshold, so noninferiority was concluded (Figure 1), and statistical superiority was supported by the CIs not crossing zero and a corresponding *P*-value of <.001.

**Figure 2.**
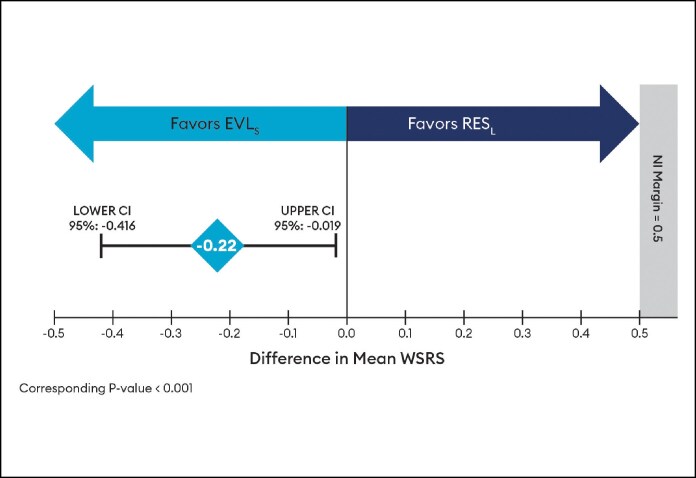
Wrinkle Severity Rating Scale difference in mean change at Month 6 by independent photographic review panel for Evolysse Smooth (EVL_S_) arm.

Similarly, for the EVL_S_ primary endpoint (*n* = 62), the absolute difference in the mean WSRS change from baseline to Month 6 for EVL_S_ vs RES_L_ was −0.22 (95% CI, −0.416 to −0.019), favoring EVL_S_, and the upper bound of the 95% CI was <0.5 threshold, so noninferiority was concluded (Figure 2), and statistical superiority was supported by the CIs not crossing zero and a corresponding *P*-value of <.001.

**Figure 3.**
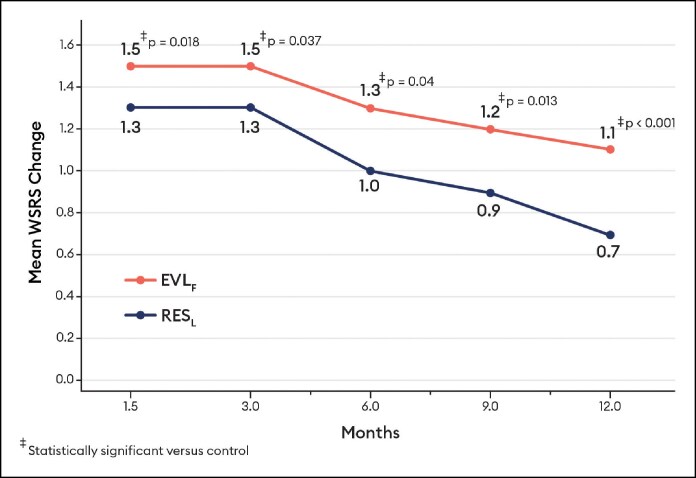
Wrinkle Severity Rating Scale mean change by blinded evaluator live assessment for Evolysse Form (EVL_F_) arm.

**Figure 4.**
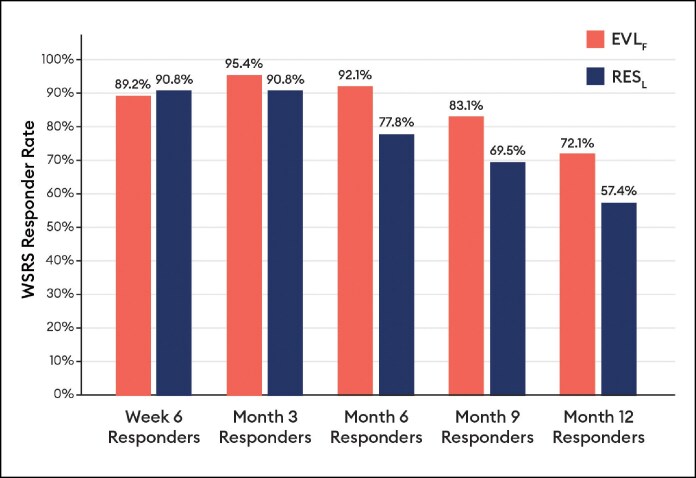
Wrinkle Severity Rating Scale responder rate by blinded evaluator live assessment for Evolysse Form (EVL_F_) arm.

**Figure 5.**
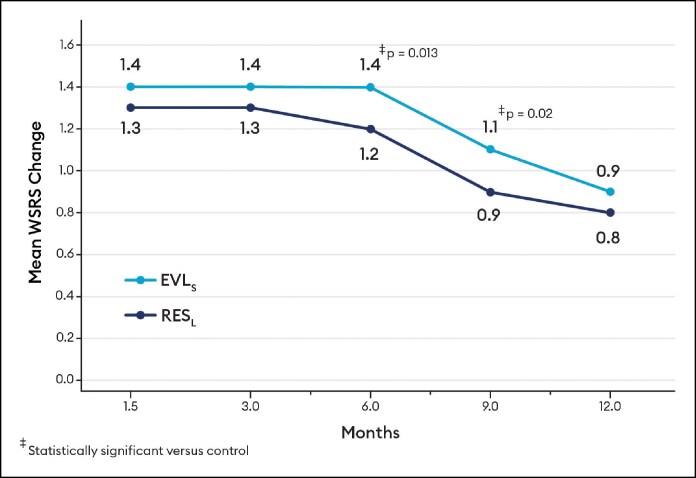
Wrinkle Severity Rating Scale mean change by blinded evaluator live assessment for Evolysse Smooth (EVL_S_) arm.

**Figure 6.**
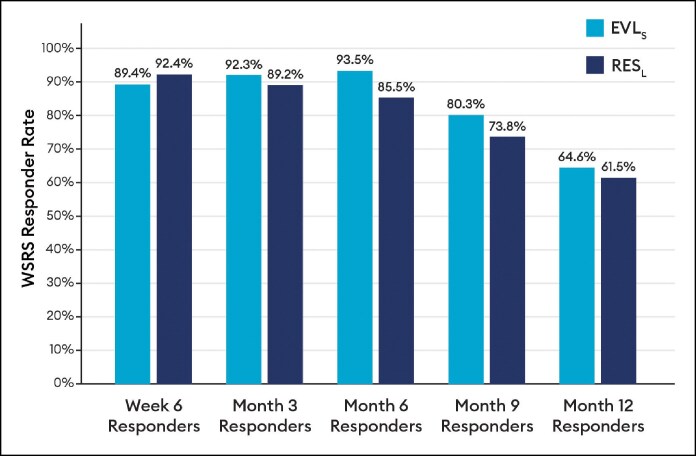
Wrinkle Severity Rating Scale responder rate by blinded evaluator live assessment for Evolysse Smooth (EVL_S_) arm.

**Figure 7.**
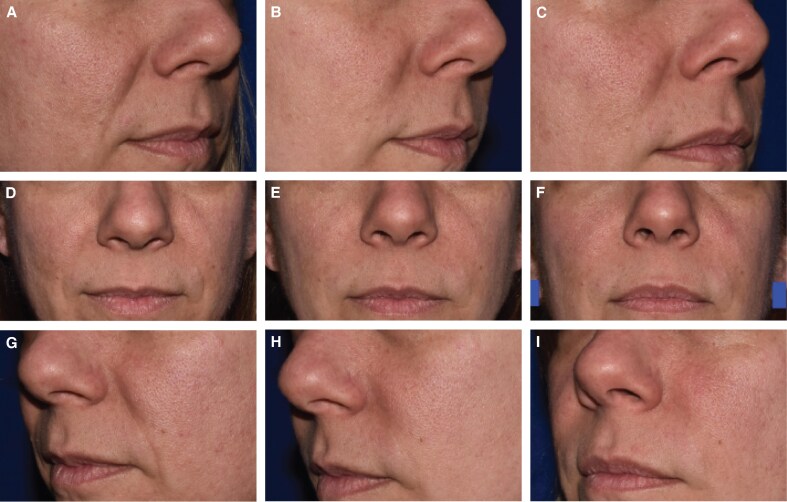
A 46-year-old White Hispanic female patient treated with 1.0 mL Evolysse Form (EVL_F_) in the left nasolabial fold (NLF) and 1.3 mL Restylane-L (RES_L_) in the right NLF at baseline (Wrinkle Severity Rating Scale [WSRS] severe), Month 6 (WSRS mild), and Month 12 (WSRS moderate). (A) Right oblique view preoperatively, (B) at Month 6, and (C) at Month 12. (D-F) Anterior view and (G-I) left oblique view at the same time intervals previously stated.

**Figure 8.**
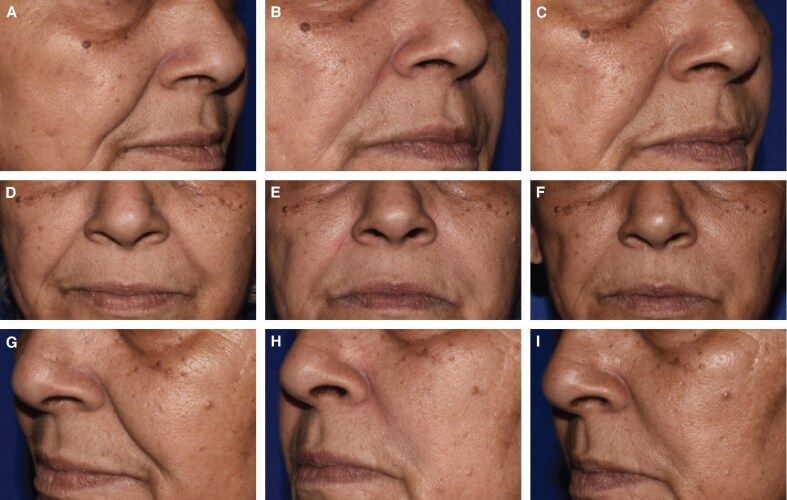
A 59-year-old White Hispanic female patient treated with 1.3 mL Evolysse Smooth (EVL_S_) in the left nasolabial fold (NLF) and 1.3 mL Restylane-L (RES_L_) in the right NLF at baseline (Wrinkle Severity Rating Scale [WSRS] severe), Month 6 (WSRS mild on left and moderate on right), and Month 9 (WSRS mild on left and moderate on right). (A) Right oblique view preoperatively, (B) at Month 6, and (C) at Month 9. (D-F) Anterior view and (G-I) left oblique view at the same time intervals previously stated.

Blinded evaluator live assessments of WSRS showed a mean change from baseline that was significantly better for EVL_F_ than RES_L_ at all visits through 12 months (Figure 3) and the vast majority of EVL_F_ NLFs remained responders throughout the study (Figure 4). EVL_S_ was significantly better than RES_L_ at 6 and 9 months (Figure 5) and the vast majority of EVL_S_ NLFs remained responders throughout the study (Figure 6). Example photographs of the lasting correction are provided in Figure 7 for an EVL_F_ patient and Figure 8 for EVL_S_ patient. Month 6 WSRS responder rates per the photographic review panel were 45.2% for EVL_F_ and 38.7% for RES_L_ and 51.6% for EVL_S_ and 35.5% for RES_L_. At all time points, blinded evaluators most commonly rated both NLFs as equivalent on the WSRS, with superior ratings more common for the EVL NLFs. For example, at Month 6 in the EVL_F_ arm, 49.2% were equivalent, 38.1% EVL_F_ superior, and 12.7% RES_L_ superior; in the EVL_S_ arm, 69.4% were equivalent, 25.8% EVL_S_ superior, and 4.8% RES_L_ superior.

Most patients were responders on the GAIS throughout the study, according to ratings by blinded evaluators (6 weeks: EVL_F_ 96.9%, RES_L_ 100%; EVL_S_ 97.0%, RES_L_ 97.0%; 12 months: EVL_F_ 73.8%, RES_L_ 60.7%; EVL_S_ 66.2%, RES_L_ 66.2%), treating investigators (6 weeks: EVL_F_ 96.9%, RES_L_ 98.5%; EVL_S_ 100%, RES_L_ 100%; 12 months: EVL_F_ 77.0%, RES_L_ 65.6%; EVL_S_ 73.8%, RES_L_ 66.2%), and patients (6 weeks: EVL_F_ 95.4%, RES_L_ 93.8%; EVL_S_ 98.5%, RES_L_ 98.5%; 12 months: EVL_F_ 80.3%, RES_L_ 72.1%; EVL_S_ 92.3%, RES_L_ 86.2%).

The FACE-Q appraisal of NLFs’ overall mean score showed significant improvement from baseline (*P* < .0001) at all time points through Month 12 for all treatment groups. Before treatment, 88.6% of patients were extremely or moderately bothered by the depth of their EVL_F_ NLFs, which improved to 31.7% at Month 6; for EVL_S_ NLFs, it was 82.9% improving to 24.2%.

On the 0 to 10 pain scale, mean injection pain for these fillers with lidocaine was 2.7 to 3.0 across the treatment groups, with all reducing to 0.2 at 30 min after injection.

Assessments of these new fillers on the Aesthetic Appearance Questionnaire showed that at Month 6 for the EVL_F_ NLFs 79.4% of patients reported that their NLF looked better overall, 84.1% that the results looked natural, 82.5% that the results felt natural, 84.1% that they would receive the treatment again, and 82.5% that they would recommend the treatment to friends. Likewise, for the EVL_S_ NLFs 83.6% of patients reported that their NLF looked better overall, 86.9% that the results looked natural, 88.5% that the results felt natural, 90.2% that they would receive the treatment again, and 87.1% that they would recommend the treatment to friends.

Subgroup analyses demonstrated that EVL_F_ and EVL_S_ are effective for moderate and severe NLFs in both men and women and all races, ethnicities, Fitzpatrick skin phototypes, and age groups, with or without touch-up treatment.

### Safety

The most common treatment responses after initial treatment for all treatment groups were injection-site swelling and tenderness (Tables 2, 3). These were also the most common responses after retreatment though the rates were lower (swelling 52.2%-54.5% with EVL_F_, 44.2%-46.5% with EVL_S_; tenderness 45.7%-52.3% with EVL_F_, 41.9%-44.2% with EVL_S_). Most treatment responses were mild or moderate severity and resolved within 1 week for all NLFs after initial treatment and retreatment.

**Table 2.** Common Treatment Responses After Initial Treatment for EVL_F_ Arm

System organ class/lowest level term	EVL_F_ (*n* = 66) *n* (%)	RES_L_ (*n* = 66) *n* (%)	Systemic (*n* = 66) *n* (%)
General disorders and administration site conditions	61 (92.4)	61 (92.4)	—
Injection-site tenderness	50 (75.8)	47 (71.2)	—
Injection-site swelling	46 (69.7)	46 (69.7)	—
Injection-site bruising	40 (60.6)	37 (56.1)	—
Injection-site redness	38 (57.6)	32 (48.5)	—
Injection-site lump	38 (57.6)	36 (54.5)	—
Injection-site pain	35 (53.0)	33 (50.0)	—
Injection-site discoloration	24 (36.4)	22 (33.3)	—
Injection-site itching	19 (28.8)	21 (31.8)	—
Facial discomfort	1 (1.5)	1 (1.5)	—
Sensation of foreign body	1 (1.5)	—	—
Nervous system disorders	1 (1.5)	1 (1.5)	4 (6.1)
Headache	—	—	4 (6.1)
Burning sensation	—	1 (1.5)	—
Facial droop	1 (1.5)	—	—
Speech impairment not otherwise specified	—	—	1 (1.5)
Eye disorders	2 (3.0)	2 (3.0)	—
Blurry vision	1 (1.5)	1 (1.5)	—
Eye pain	—	1 (1.5)	—
Eyelid twitching	1 (1.5)	—	—
Skin and subcutaneous tissue disorders	2 (3.0)	—	—
Petechiae	1 (1.5)	—	—
Puffy skin	1 (1.5)	—	—
Blood and lymphatic system disorders	1 (1.5)	—	—
Lymph node pain	1 (1.5)	—	—
Musculoskeletal and connective tissue disorders	—	1 (1.5)	—
Jaw pain	—	1 (1.5)	—

EVL_F_, Evolysse Form; RES_L_, Restylane-L.

**Table 3.** Common Treatment Responses After Initial Treatment for EVL_S_ Arm

System organ class/lowest level term	EVL_S_ (*n* = 64) *n* (%)	RES_L_ (*n* = 64) *n* (%)	Systemic (*n* = 64) *n* (%)
General disorders and administration site conditions	53 (82.8)	53 (82.8)	—
Injection-site swelling	36 (56.3)	40 (62.5)	—
Injection-site tenderness	41 (64.1)	42 (65.6)	—
Injection-site bruising	33 (51.6)	35 (54.7)	—
Injection-site lump	34 (53.1)	38 (59.4)	—
Injection-site pain	32 (50.0)	34 (53.1)	—
Injection-site redness	33 (51.6)	32 (50.0)	—
Injection-site discoloration	26 (40.6)	25 (39.1)	—
Injection-site itching	18 (28.1)	22 (34.4)	—
Facial swelling	1 (1.6)	1 (1.6)	—
Injection-site dryness	1 (1.6)	1 (1.6)	—
Eye disorders	2 (3.1)	3 (4.7)	—
Blurry vision	1 (1.6)	1 (1.6)	—
Double vision	1 (1.6)	1 (1.6)	—
Eye pain	1 (1.6)	1 (1.6)	—
Eyes heavy feeling of	—	1 (1.6)	—
Nervous system disorders	—	—	2 (3.1)
Headache	—	—	2 (3.1)
Product issues	1 (1.6)	—	—
Migration of implant	1 (1.6)	—	—
Skin and subcutaneous tissue disorders	1 (1.6)	1 (1.6)	—
Acne	1 (1.6)	1 (1.6)	—
Skin discoloration	1 (1.6)	1 (1.6)	—
Vascular disorders	1 (1.6)	1 (1.6)	—
Flushed face	1 (1.6)	1 (1.6)	—

EVL_S_, Evolysse Smooth; RES_L_, Restylane-L.

In the EVL_F_ arm, there were treatment-related AEs in 11 EVL_F_ NLFs (15.7%) and 8 RES_L_ NLFs (11.4%) after initial treatment, most commonly injection-site mass (8.6% for EVL_F_, 2.9% for RES_L_), with pain, swelling, pruritus, discomfort, discoloration, bruising, inflammation, nodule, jaw pain, and foreign body sensation occurring in 1 or 2 patients each. After retreatment, 1 patient had injection-site pain in each NLF, and 1 had a headache. All treatment-related AEs were mild severity except for 1 moderate severity jaw pain for RES_L_, and all resolved. There were 2 delayed-onset AEs that occurred in RES_L_ NLFs 6 months after touch-up treatment, and both of these required treatment. One patient received oral antihistamines and topical corticosteroids for injection-site inflammation, and 1 patient received oral antibiotics for injection-site nodule; both resolved within 3 weeks after treatment.

In the EVL_S_ arm, there were treatment-related AEs in 5 EVL_S_ NLFs (7.1%) and 6 RES_L_ NLFs (8.6%) after initial treatment, most commonly injection-site discoloration (4.3%), injection-site mass (2.9% for EVL_S_, 4.3% for RES_L_), and injection-site erythema (2.9%) in both NLFs, with pain, swelling, pruritus, bruising, flushing, and papule occurring in just a single patient each. After retreatment there was 1 bilateral injection-site bruising and 1 each unilateral injection-site mass, injection-site pain, and injection-site swelling. All treatment-related AEs were mild or moderate severity and resolved.

There were no treatment-related serious AEs and no changes in extraocular motility or confrontation visual fields throughout the study. There was 1 patient with a transient decrease in visual acuity from 20/25 at screening to 20/30 at Week 6 and a return to 20/25 at Month 3 and all subsequent time points.

Because of the small sample size, safety data are limited for Fitzpatrick skin Types V and VI for EVL_F_. However, subgroup analyses supported safety across subgroups by sex, race, ethnicity, age group, Fitzpatrick skin type, injection volume, baseline WSRS score, touch-up status, and needle size.

## DISCUSSION

With the results of this study, the authors showed that the new cold-crosslinked fillers are safe and effective for correction of NLFs. The primary endpoints were met, demonstrating that EVL_F_ and EVL_S_ were both noninferior to RES_L_ and further that they were statistically superior according to evaluations by an independent photographic review panel. Blinded evaluator live WSRS ratings showed that the vast majority of EVL_F_ and EVL_S_ NLFs remained responders throughout the 12-month follow-up period, with mean change from baseline that was significantly better than RES_L_ at all visits through 12 months for EVL_F_ (despite similar injection volumes: mean 1.2 mL for EVL_F_ and 1.3 mL for RES_L_) and at 6 and 9 months for EVL_S_ (despite lower injection volume mean of 1.0 mL for EVL_S_ and 1.2 mL for RES_L_). Safety assessments showed that all of the fillers were safe and tolerable, with expected treatment responses and AEs for filler injections. As anticipated for fillers containing lidocaine, procedural pain was minimal for the new fillers and the control filler.

The blinded evaluator live WSRS responder rates were substantially higher than those from the independent photographic review panel, but this finding is consistent with other filler studies. A study of Juvéderm Voluma XC (Allergan Aesthetics, an AbbVie company, Irvine, CA) had Month 6 responder rates on the validated Allergan Chin Retrusion Scale of 91.8% for live assessments vs 56.3% for photographic assessments.^5^ Similarly, a study of RES_L_ and Juvéderm Volbella XC (Allergan Aesthetics, an AbbVie company) had Month 3 responder rates on the validated Allergan Lip Fullness Scale of 81.4% and 84.2%, respectively, for live assessments vs 29.3% and 34.1%, respectively, for photographic assessments. This is not unexpected given that photographic images provide less detail than live evaluations.^6^

In an earlier randomized, controlled split-face European study,^7^ 45 patients were treated with EVL_F_ and RES_L_ in their NLFs, and the primary endpoint of mean WSRS change at 1 month by blinded evaluator demonstrated noninferiority of EVL_F_ as well as statistical superiority (*P* < .001). Furthermore, secondary endpoints showed significantly better mean WSRS scores for EVL_F_ than RES_L_ at 3 months (*P* = .018) and 6 months (*P* = .037). Objective measurements of wrinkle volume with the fringe projection system DermaTOP (Eotech, France) showed that despite the same mean injection volume for both products, the NLF volume was significantly better for EVL_F_ at 6 months (*P* = .038). On the GAIS, patient ratings showed similar results for both products, whereas blinded evaluator ratings showed significantly better results for EVL_F_ at 3 and 6 months. Both products resulted in mild-to-moderate transient injection-site responses and AEs.

EVL_S_ was evaluated in a previous prospective, multicenter, open-label study^8^ in which 72 patients received a single treatment with another cold-crosslinked HA filler (ESTYME LIPS, Symatese, France) in their lips, and 61 patients also received EVL_S_ in their NLFs and lip fine lines. On 6-point validated scales, mean NLF improvement ranged from 1.3 points at 30 days to 0.7 points at 12 months, and mean lip fine lines improvement ranged from 1.6 points at 30 days to 1 point at 12 months. A majority of patients were responders at all time points through 12 months on the validated scales as well as investigator and patient ratings on the GAIS. Injection-site responses and AEs were primarily of mild severity.

Limitations of this study include a comparison with only a single HA injectable control, recognizing that this is standard for FDA registration studies, and time to complete loss of correction was not established as the majority of EVL_F_ and EVL_S_ NLFs continued to show improvement at the end of the study.

## CONCLUSIONS

The new cold-crosslinked fillers were shown to be safe and effective for correction of NLFs, with results lasting for 1 year.
